# Associations between body composition and nutritional assessments and biochemical markers in patients with chronic radiation enteritis: a case–control study

**DOI:** 10.1186/s12937-016-0177-6

**Published:** 2016-05-28

**Authors:** Zhongliang CAI, Da CAI, Danhua YAO, Yong CHEN, Jian WANG, Yousheng LI

**Affiliations:** 1 Department of Surgery, Nanjing Clinical College of the Secondary Military Medical University, Nanjing, Jiangsu 210002 China; 2 Department of Radiology, Hangzhou Sanatorium of Nanjing Command, PLA, Hangzhou, Zhejiang 310007 China; 3 Department of Surgery, Jinling hospital, 305 East Zhongshan Road, Nanjing, Jiangsu 210002 China

**Keywords:** Body composition, Nutritional assessment, Biochemical markers, Chronic radiation enteritis

## Abstract

**Background:**

Chronic radiation enteritis (CRE) is defined as loss of absorptive capacity after irradiation due to chronic inflammation and damage of intestinal mucosa, which may lead to varying degrees of malnutrition. The aim of this study was to evaluate the potential correlation between the nutritional status and systemic inflammation in patients with CRE.

**Methods:**

Medical records of 92 patients with CRE and 184 age- and sex-matched controls in a single center from January 2010 to October 2015 were retrospectively reviewed. All enrolled subjects underwent nutritional status analysis, including three different nutritional indices: Nutritional Risk Screening-2002 (NRS-2002), Patient-generated Subjective Global Assessment (PG-SGA) and Controlling Nutritional Status (CONUT), bioelectrical impedance spectroscopy (BIS), and biochemical markers, within 24 h of admission.

**Results:**

The results showed that NRS-2002, PG-SGA and CONUT were all positively correlated with neutrophil/lymphocyte ratio (NLR) (*r* = 0.304, 0.384 and 0.425, all *p* < 0.001) and C-reactive protein (CRP) (*r* = 0.357, 0.479 and 0.230, all *p* < 0.001), while negatively correlated with albumin (*r* = −0.612, −0.727 and −0.792, all *p* < 0.001) and total cholesterol (TC) (*r* = −0.485, −0.545 and −0.473, all *p* < 0.001) in patients with CRE, respectively. Body cell mass (BCM) has been deemed a key body composition parameter. It was positively correlated with albumin (*r* = 0.489, *p* < 0.001) and TC (*r* = 0.237, *p* < 0.001), while negatively correlated with NLR (*r* = −0.140, *p* = 0.02) and CRP (*r* = −0.215, *p* < 0.001). A multivariate linear regression analysis showed that values of intracellular water (*β* coefficient = 0.760, *p <* 0.001), extracellular water (*β* coefficient = 0.006, *p =* 0.011), protein (*β* coefficient = 0.235, *p <* 0.001) and CRP (*β* coefficient = 0.001, *p =* 0.009) were independent determinants of BCM.

**Conclusion:**

This study revealed that BIS combined with nutritional assessments and biochemical markers were appropriate methods to assess the nutritional and inflammatory status in patients with CRE. Furthermore, the nutritional status was verified to be significantly correlated with systemic inflammation.

## Background

Radiation enteritis (RE) is defined as inflammation and damage of intestinal mucosa by short exposure to radiation at the abdomen and pelvis level, which can be subdivided into acute (ARE) and chronic radiation enteritis (CRE). ARE mostly occurs days or weeks after radiation therapy and CRE may emerge after several years or even decades [1, 2]. CRE results from obliterative endarteritis which progresses to intestinal wall thickening, ulceration and fibrosis, leading to intestinal stricture, fistula and even perforation. Because of insufficient intestinal mucosa for nutrition absorption, most CRE patients suffer from mild, moderate or severe malnutrition [3]. The volume of small bowel affected and total radiation dose are the most important factors of the risks of acute and late toxicity. Acute inflammation may resolve and develop into a more chronic state with arteriolar endarteritis and fibrosis. This progressive vasculitis causes intestinal ischemia that in turn leads to mucosal friability and neovascularization as well as an exaggerated submucosal fibrosis.

According to the consensus updated, nutrition diagnoses could be categorized into three species: 1. Starvation-related malnutrition, which is chronic malnutrition without inflammation; 2. Chronic disease-related malnutrition, where inflammation is chronic and of mild to moderate degree; 3. Acute disease and injury-related malnutrition, where inflammation is acute and severe [4, 5]. Irrespective of disease severity, inflammation always plays a vital role in malnutrition [6, 7]. Based on the aforementioned nutritional definitions, the nutritional status of CRE is presumed to be classified as chronic disease-related malnutrition, where inflammation is chronic and of mild to moderate degree.

Evaluation of the correlation between the nutritional status and systemic inflammation was well conducted in chronic undernourished patients [8]. Nevertheless its effect has not yet been validated in patients with CRE. The objective of this study was to evaluate the potential correlation between the nutritional status and systemic inflammation in patients with CRE.

## Methods

### Protocol and participants

Clinical records of patients admitted to our center with CRE as a definite diagnosis from January 2010 to October 2015 were retrospectively reviewed. The diagnoses depended on the combination of clinical symptoms, medical histories and the exclusion of other potential diagnoses. Clinical manifestations included weight loss, abdominal pain, rectal bleeding, diarrhea, malabsorption, stricture, intestinal obstruction and even atraumatic perforation [1, 2, 9]. Gastrointestinal endoscopy was most useful in excluding other causes such as infection and recurrent neoplasia [9]. Moreover, imaging scans have been used to support a suspected diagnosis. Magnetic resonance enterography has shown focal abnormalities generally in the distal ileum of CRE patients [10].

Most patients presented with symptoms of partial intestinal obstruction, bleeding or fistula at least 6 months after radiotherapy. Given the poor nutritional status, preoperative nutrition support by parenteral nutrition or enteral nutrition was provided. All patients underwent body composition analysis assessed by bioelectrical impedance spectroscopy (BIS) before surgery. Patients were matched 1:2 by age and sex with healthy people who had the medical examination in our center. Patients with ARE, tumor recurrence and concomitant chronic diseases such as diabetes, chronic renal failure and liver cirrhosis were excluded. Furthermore, patients with incomplete information due to physical, cognitive or mental problems were also excluded. Data regarding nutritional assessments, BIS and biochemical markers were extracted for stepwise analysis. The baseline demographic characteristics of subjects enrolled were also reviewed.

The study was conducted in accordance with the Declaration of Helsinki and approved by the Institutional Ethical Committee of Jinling hospital. All patients gave their informed consent prior to entering the study.

### Nutritional assessments

Nutritional Risk Screening-2002 (NRS-2002) Questionnaire was scored in each of three components, including nutritional status, severity of disease and age [11]. Patient-Generated Subjective Global Assessment (PG-SGA) comprised of two sections that were completed by patients and clinicians, respectively. Patients were required to fulfill the form included weight loss, intake, symptoms and functional capacity initially. Afterwards, clinicians assessed the disease status and its relation with metabolic demands, nutritional requirements and physical examinations. Patients were classified based on a total score of two sections summed and a higher score reflected a higher risk of malnutrition [12]. The Controlling Nutritional Status (CONUT) score was based on the method developed by Ulibarri et al. [13]. The score was calculated by three parameters, including serum albumin, total cholesterol level and total lymphocyte count. All these nutritional assessments were implemented within the first 24 h of hospital admission and the results were analyzed retrospectively.

### Anthropometric measurements and body composition analysis

Body composition was measured by BIS, a selective approach of bioelectrical impedance analysis (BIA). When weight and height are measured, patients were required to run the test after meal for at least 2 h with barefoot and underwear. Body mass index (BMI) was calculated as on weight in kilogram divided by height in meters squared (kg/m^2^). Then the patient lay down fully relaxed with limbs abducted from the body. After removing electrodes with alcohol cotton, attach them at each hand wrist and foot ankle to make sure the electrode contact area is beyond 5 cm^2^. The instrument used was multi-frequency electrical impedance analyzer (Inbody S20, Korea) with frequency ranging from 1 kHz to 1 MHz and 800A constant electrical flow through the body to measure the impedance value. The whole process took only less than 2 min. All data were stored within the instrument and analyzed by computer software automatically. Body composition parameters including fat-free mass (FFM), fat mass (FM), body cell mass (BCM), total body water (TBW), extracellular water (ECW), intracellular water (ICW), protein and mineral were collected.

### Biochemical markers analysis

Blood samples (5 mL) were collected from a peripheral vain with a single puncture early in the morning. Biochemical markers included albumin, neutrophil count, lymphocyte count, C-reactive protein (CRP) and total cholesterol (TC) were measured using an automated biochemical analyzer (Olympus AU400 Chemistry Analyzer, Tokyo, Japan). Neutrophil/lymphocyte ratio (NLR) was calculated from these parameters.

### Statistical analysis

Statistical analysis was performed using SPSS version 20.0 (SPSS, Inc, an IBM Company, Chicago, IL). The results of analysis were expressed as mean ± SD. Data regarding patients with CRE and matched controls were compared with student’s t test. The Pearson’s product–moment correlation coefficient was used to assess correlation of two variables. A multivariate linear regression analysis with stepwise selection of covariates was used to explore the correlation between BCM and other variables. Only factors that were statistical different could be selected into the stepwise multivariate regression analysis. All candidate variables were checked to see if their significance has been reduced below the specific level. If a non-significant variable was found, it is removed from the analysis. Both *p* < 0.01 and *p* < 0.05 was recognized as statistically significant. The compound bar chart was used to describe body composition parameters of patients compared with controls. The scatter plot was used to describe correlations between BCM and biochemical markers in patients with CRE.

## Results

### Demographic characteristics, nutritional assessments and biochemical markers

**Fig. 1:**
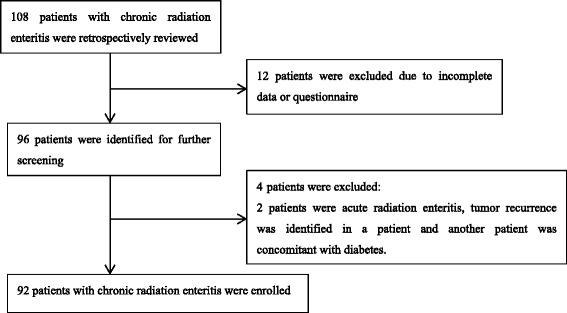
Flow chart of patients who met inclusion/exclusion criteria for the study population

A total of 92 patients and 184 controls were identified and enrolled in this study. The detailed flow chart of patients with CRE who met inclusion/exclusion criteria for the study population was presented in Fig. 1. As expected, there were no differences between the two groups in terms of age (53 ± 11.54 *vs.* 52.98 ± 11.51 years, *p* = 0.998) and height (161.96 ± 8.06 *vs.* 161.21 ± 7.28 cm, *p* = 0.440), while the results showed that patients with CRE had lower values of body weight (45.68 ± 8.68 *vs.* 63.79 ± 11.55 kg, *p* < 0.001) and BMI (17.39 ± 2.67 *vs.* 24.44 ± 3.42 kg/m^2^, *p* < 0.001) than those in the healthy control group. Nutritional assessments included NRS-2002 (2.45 ± 0.86 *vs.* 0.26 ± 0.69, *p* < 0.001), PG-SGA (13.75 ± 4.08 *vs.* 1.24 ± 0.65, *p* < 0.001) and CONUT (2.05 ± 1.91 *vs.* 0, *p* < 0.001) demonstrated that patients had poorer nutritional status than controls. Biochemical markers included albumin (36.61 ± 5.56 *vs.* 46.40 ± 2.58 g/L, *p* < 0.001), NLR (3.36 ± 2.28 *vs.* 1.84 ± 0.78, *p* < 0.001), CRP (11.32 ± 9.98 *vs.* 2.77 ± 3.80 mg/L, *p* < 0.001) and TC (3.70 ± 0.99 *vs.* 5.25 ± 1.08 mmol/L, *p* < 0.001) were also compared. Demographic characteristics, nutritional assessments and biochemical markers of patients with CRE compared with controls are shown in Table 1.

**Table 1:** Demographic characteristics, nutritional assessments and biochemical indices of patients with CRE compared with controls

	CRE (*n* = 92)	Control (*n* = 184)	*P* value
Age (years)	53 ± 11.54	52.98 ± 11.51	0.998
Sex	65 females/27 males	130 females/54 males	
Height (cm)	161.96 ± 8.06	161.21 ± 7.28	0.440
Weight (kg)	45.68 ± 8.68	63.79 ± 11.55	<0.001
BMI (kg/m^2^)	17.39 ± 2.67	24.44 ± 3.42	<0.001
NRS-2002	2.55 ± 0.97	0.26 ± 0.69	<0.001
PG-SGA	14.18 ± 3.25	2.01 ± 0.82	<0.001
CONUT	2.05 ± 1.91	0	<0.001
Albumin (g/L)	36.61 ± 5.56	46.40 ± 2.58	<0.001
NLR	3.36 ± 2.28	1.84 ± 0.78	<0.001
CRP (mg/L)	11.32 ± 9.98	2.77 ± 3.80	<0.001
TC (mmol/L)	3.70 ± 0.99	5.25 ± 1.08	<0.001

*BMI* body mass index, *NRS-2002* Nutritional Risk Screening-2002, *PG-SGA* Patient-Generated Subjective Global Assessment, *CONUT* Controlling Nutritional Status, *NLR* neutrophil/lymphocyte ratio, *CRP* C-reactive protein, *TC* total cholesterol

### Body composition analysis

**Fig. 2:**
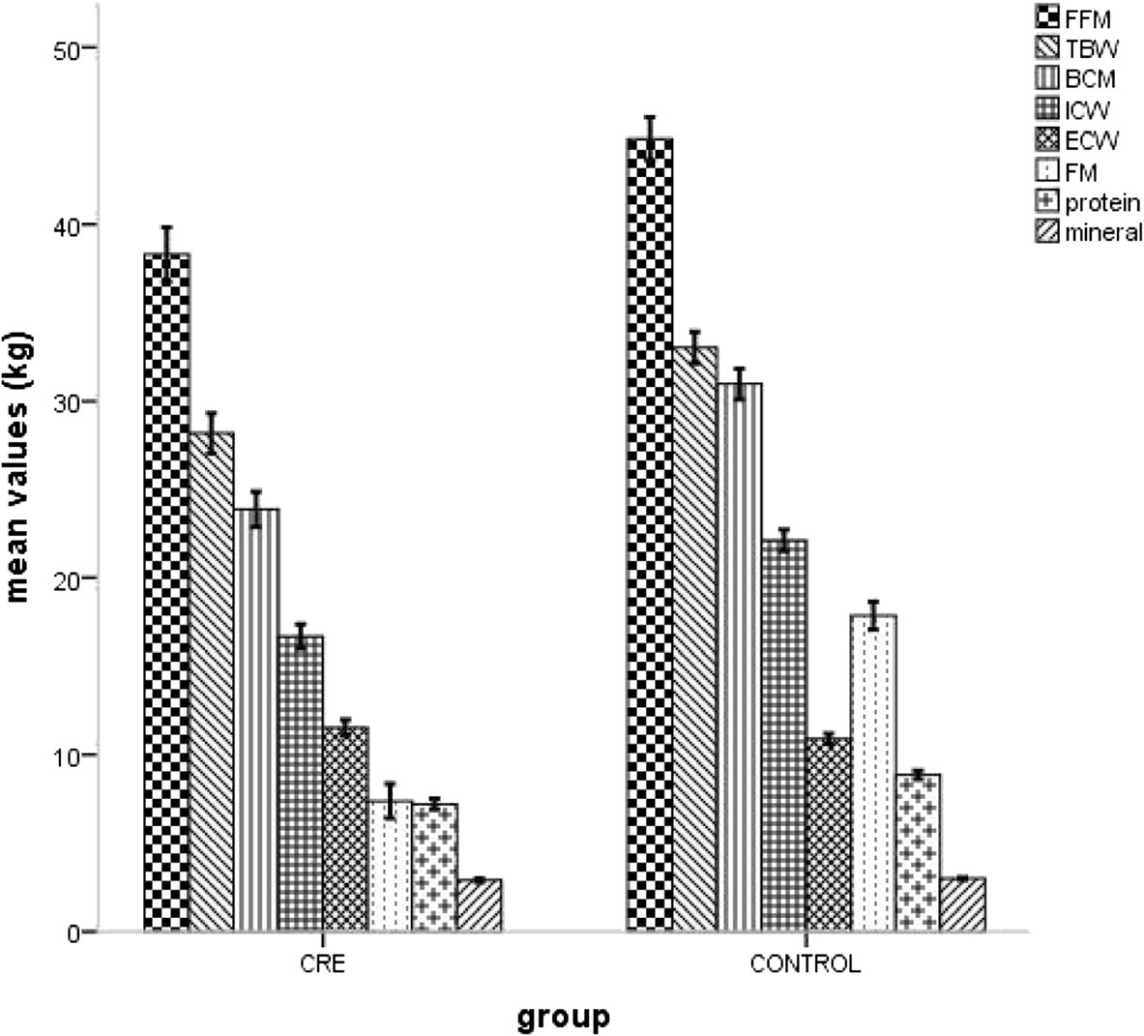
Body composition parameters of patients with CRE compared with controls. CRE: chronic radiation enteritis; ICW: intracellular water; ECW: extracellular water; TBW: total body water; FFM: fat-free mass; FM: fat mass; BCM: body cell mass

The results demonstrated that patients with CRE had significantly lower values of FFM (38.18 ± 7.38 *vs.* 45.50 ± 8.43 kg, *p* < 0.001), FM (7.45 ± 4.77 *vs.* 18.30 ± 5.64 kg, *p* < 0.001), BCM (23.86 ± 4.77 *vs.* 31.42 ± 5.99 kg, *p* < 0.001), ICW (16.66 ± 3.34 *vs.* 22.44 ± 4.28 kg, *p* < 0.001), TCW (28.13 ± 5.41 *vs.* 33.51 ± 6.26 kg, *p* < 0.001), protein (7.16 ± 1.48 *vs.* 8.98 ± 1.71 kg, *p* < 0.001), mineral (2.88 ± 0.64 *vs.* 3.01 ± 0.45 kg, *p* = 0.048) and a slightly higher value of ECW (11.51 ± 2.25 vs. 11.06 ± 2.05 kg, *p* = 0.094) than those in the control group (see Table 2 and Fig. 2).

**Table 2:** Body composition parameters of patients with CRE compared with controls

	CRE (*n* = 92)	Control (*n* = 184)	*P* value
ICW (kg)	16.66 ± 3.34	22.44 ± 4.28	<0.001
ECW (kg)	11.51 ± 2.25	11.06 ± 2.05	0.094
TBW (kg)	28.13 ± 5.41	33.51 ± 6.26	<0.001
FFM (kg)	38.18 ± 7.38	45.50 ± 8.43	<0.001
FM (kg)	7.45 ± 4.77	18.30 ± 5.64	<0.001
BCM (kg)	23.86 ± 4.77	31.42 ± 5.99	<0.001
Protein (kg)	7.16 ± 1.48	8.98 ± 1.71	<0.001
Mineral (kg)	2.88 ± 0.64	3.01 ± 0.45	0.048

*CRE* chronic radiation enteritis, *ICW* intracellular water, *ECW* extracellular water, *TBW* total body water, *FFM* fat-free mass, *FM* fat mass, *BCM* body cell mass

### Correlations between nutritional assessments and biochemical markers in patients with CRE

Nutritional assessments included NRS-2002, PG-SGA and CONUT were all positively correlated with NLR (*r* = 0.304, 0.384 and 0.425, all *p* < 0.001) and CRP (*r* = 0.357, 0.479 and 0.230, all *p* < 0.001), while negatively correlated with albumin (*r* = −0.612, −0.727 and −0.792, all *p* < 0.001) and TC (*r* = −0.485, −0.545 and −0.473, all *p* < 0.001) respectively (see Table 3).

**Table 3:** Correlations between nutritional assessments and biochemical markers in patients with CRE

		Albumin	NLR	CRP	TC
NRS-2002	*r*	−0.612**	0.304**	0.357**	−0.485**
	*p*	<0.001	<0.001	<0.001	<0.001
PG-SGA	*r*	−0.727**	0.384**	0.479**	−0.545**
	*p*	<0.001	<0.001	<0.001	<0.001
CONUT	*r*	−0.792**	0.425**	0.230**	−0.473**
	*p*	<0.001	<0.001	<0.001	<0.001

**Correlation is significant at the 0.01 level. *CRE* chronic radiation enteritis, *NLR* neutrophil/lymphocyte ratio, *CRP* C-reactive protein, *TC* total cholesterol, *NRS-2002* Nutritional Risk Screening-2002, *PG-SGA* Patient-Generated Subjective Global Assessment, *CONUT* Controlling Nutritional Status

### Correlations between body composition parameters and biochemical markers in patients with CRE

**Fig. 3:**
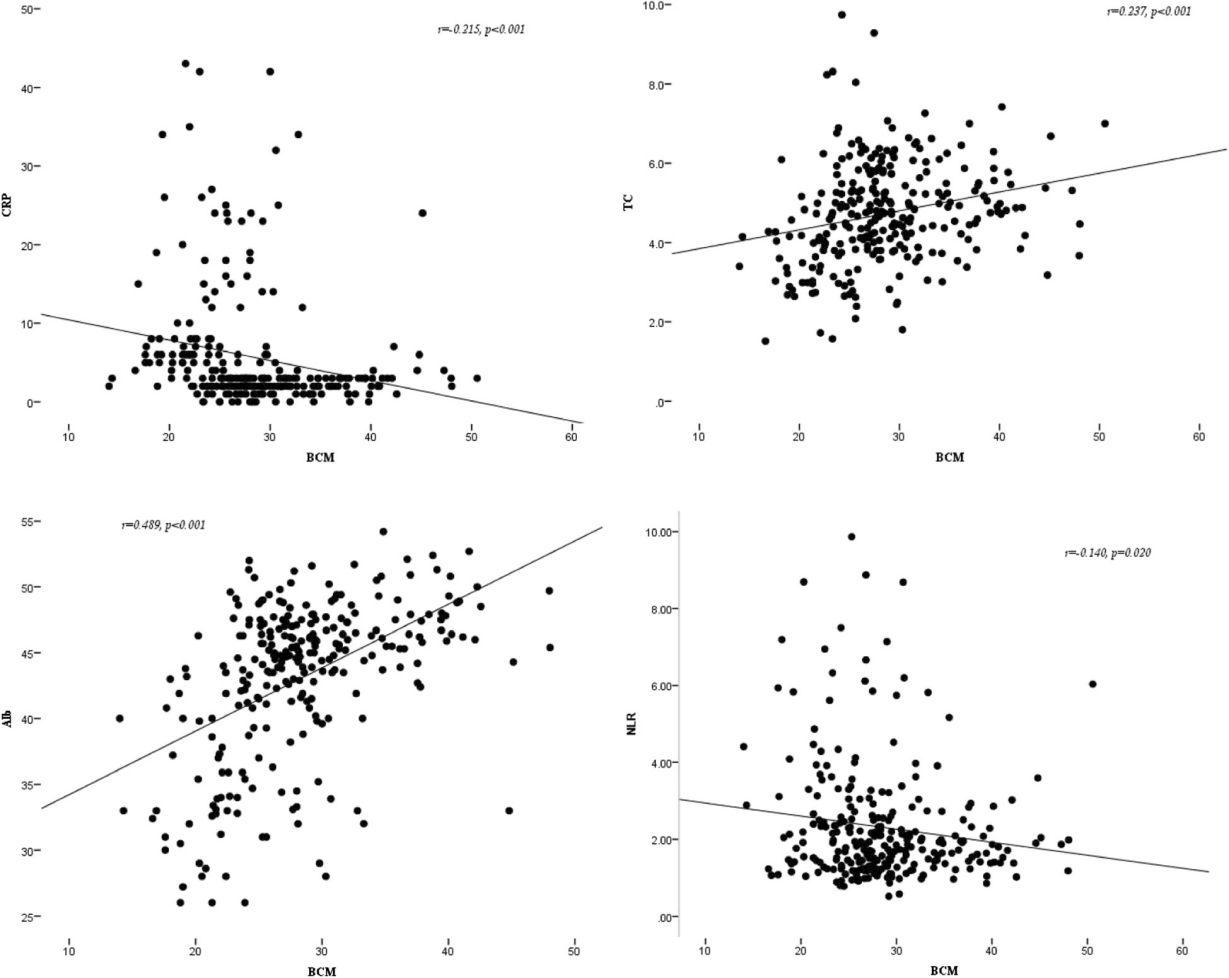
Scatter plots describing correlations between BCM and biochemical markers in patients with CRE. Correlation is significant at the 0.05 level. CRE: chronic radiation enteritis; BCM: body cell mass; CRP: C-reactive protein; NLR: neutrophil/lymphocyte ratio; TC: total cholesterol

BCM, a key body composition parameter, was positively correlated with albumin (*r* = 0.489, *p* < 0.001) and TC (*r* = 0.237, *p* < 0.001), while negatively correlated with NLR (*r* = −0.140, *p* = 0.02) and CRP (*r* = −0.215, *p* < 0.001). Scatter plots describing correlations between BCM and biochemical markers are shown in Fig. 3. Correlations between other body composition parameters and biochemical markers are also documented in Table 4.

**Table 4:** Correlations between body composition parameters and biochemical markers in patients with CRE

		Albumin	NLR	CRP	TC
BCM	*r*	0.489**	−0.140*	−0.215**	0.237**
	*p*	<0.001	0.020	<0.001	<0.001
ICW	*r*	0.511**	−0.155*	−0.233**	0.255**
	*p*	<0.001	0.010	<0.001	<0.001
TBW	*r*	0.349**	−0.077	−0.124*	0.136*
	*p*	<0.001	0.203	0.041	0.024
ECW	*r*	−0.082	0.115	0.145*	−0.158**
	*p*	0.183	0.059	0.016	0.009
FFM	*r*	0.354**	−0.074	−0.127*	0.138*
	*p*	<0.001	0.223	0.036	0.023
FM	*r*	0.585**	−0.267**	−0.375**	0.485**
	*p*	<0.001	<0.001	<0.001	<0.001
Protein	*r*	0.426**	−0.100	−0.169**	−0.188**
	*p*	<0.001	0.099	0.005	0.002
Mineral	*r*	0.116	0.072	−0.014	−0.040
	*p*	0.059	0.236	0.814	0.507

*Correlation is significant at the 0.05 level. **Correlation is significant at the 0.01 level. *CRE* chronic radiation enteritis, *NLR* neutrophil/lymphocyte ratio, *CRP* C-reactive protein, *TC* total cholesterol, *ICW* intracellular water, *ECW* extracellular water, *TBW* total body water, *FFM* fat-free mass, *FM* fat mass, *BCM* body cell mass

### Correlations between BCM and multiple variables

According to a multivariate linear regression analysis, values of ICW (*β* coefficient = 0.760, *p <* 0.001), ECW (*β* coefficient = 0.006, *p =* 0.011), protein (*β* coefficient = 0.235, *p <* 0.001) and CRP (*β* coefficient = 0.001, *p =* 0.009) were significantly and independently correlated with BCM (see Table 5).

**Table 5:** A multivariate linear regression analysis of BCM

	*β*	*P* value
Age (years)	0.001	0.155
BMI (kg/m^2^)	0.009	0.078
NRS-2002	0.001	0.164
PG-SGA	<0.001	0.905
CONUT	−0.001	0.281
FM (kg)	−0.006	0.092
ICW (kg)	0.760**	<0.001
ECW (kg)	0.006*	0.011
protein (kg)	0.235**	<0.001
mineral (kg)	−0.001	0.446
Albumin (g/L)	−0.001	0.384
NLR	0.001	0.088
CRP (mg/L)	0.001**	0.009

*Correlation is significant at the 0.05 level. **Correlation is significant at the 0.01 level. *NRS-2002* Nutritional Risk Screening-2002, *PG-SGA* Patient-Generated Subjective Global Assessment, *CONUT* Controlling Nutritional Status, *BCM* body cell mass, *FM* fat mass, *ICW* intracellular water, *ECW* extracellular water, *NLR* neutrophil/lymphocyte ratio, *CRP* C-reactive protein

## Discussion

The present study revealed that values of body composition parameters and all nutritional assessment scores were significantly lower when compared to the control population. Moreover, a bivariate and multivariate linear regression analysis revealed that nutritional assessments and body composition were significantly correlated with biochemical markers, indicating that the nutritional status was heavily associated with systemic inflammation in patients with CRE.

In patients with CRE, the extent of malnutrition depends on the degree of tissue damage together with the site of injury [14]. According to our experiences, perioperative nutrition support should be considered and laparoscopic surgery is superior to open surgery for treatment of radiation enteritis-induced intestinal stenosis [15, 16]. Resection or bypass of the affected bowel may ultimately lead to short bowel syndrome, which further compromises the nutritional status of patients [17].

Although malnutrition is acknowledged as a common concomitant disease under many circumstances, there is still a lack of universal definition and method for nutritional assessment. NRS-2002, PG-SGA and CONUT are all validated approaches that are successfully applied as nutritional assessment tools in the clinical setting [10–12]. The present study revealed that most patients with CRE suffered from malnutrition. The patients were enduring chronic malnutrition with substantial weight loss in the preceding months or year and partially food-intolerant even when severe complications occurred. With respect to biochemical markers, it is important to be aware that albumin is insufficient to assess nutritional status alone and its reduced synthesis is more related with inflammation [18]. NLR, a simple and useful method, has emerged to represent subclinical low-grade inflammation under many circumstances [19, 20]. Hypercholesterolemia was also reported to be caused by inflammation in older patients [21]. With respect to the relationship between nutritional assessments and biochemical markers, the present study demonstrated that serum albumin level was strongly correlated with NRS-2002, PG-SGA and CONUT scores, while others were not. This could be in a large part due to serum albumin is a more sensitive marker, reflecting both chronic malnutrition and inflammation in CRE patients.

Body composition has always been an important index of nutritional status. The traditional parameters for assessing body composition include body weight, muscular measurement, triceps skinfold-thickness and BMI [22]. In recent years, several methods such as imaging techniques CT and MRI have been applied for body composition analysis. However, their high cost and inconvenience for bedridden patients limit their utility in the clinical practice. Dural-energy X-ray absorptiometry (DXA) is another method which can directly assess body composition but it is also restricted for its radiation exposure and inaccessibility. Bioelectrical impedance analysis (BIA), however, is a non-invasive, cheap and convenient method to indirectly evaluate body composition of patients and is widely endorsed by doctors and patients in clinical practice [23, 24]. To date, plenty of studies have validated the method of BIA in various clinical populations. Based on the spectrum of frequencies, BIA can be divided into three approaches as single-frequency BIA (SF-BIA), multi-frequency BIA (MF-BIA) and BIS. Selectively, BIS is a solid field method superior to other approaches as it measures impedance over the entire spectrum of frequencies, rather than being limited to only 1 (in the case of SF-BIA) or 2 (in MF-BIA) frequencies [25].

The main components of body composition are FFM and FM. It was noted that the disease could cause both lean body weight and fat tissue loss in the present study, which was also verified in other chronic diseases such as chronic obstructive pulmonary disease [26]. BCM refers to intracellular compartments, which can be approximately speculated by ICW. BCM is also assumed to be of clinical relevance as the total mass of metabolically active, functioning cells and deemed a key parameter for body composition analysis [25]. The present study also demonstrated that patients with CRE had lower values of ICW and TBW but a slightly higher value of ECW than those in the control group. It was speculated that capillary-interstitial and protein leak caused by chronic inflammation occurred in patients with CRE. Mineral and protein were often underappreciated for nutritional assessment. Negative protein balance occurs commonly in the context of malnutrition. Essential minerals such as calcium, iron, cooper and zinc are all indispensable for cell signaling or electron transport and lack of them may lead to devastating consequences [27]. The present study indicated that values of BCM, ICW and FM were statistically correlated with all biochemical markers. Further analysis revealed that values of ICW, ECW, protein and CRP may explain the significant difference of BCM between CRE patients and the control populations. Thus, it could be seen that fluid and fat distribution, in particular, were the most important risk factors in CRE patients. Moreover, correlation between CRP level and body composition indicated that inflammation may be a powerful predictor of malnutrition in CRE patients.

This is the first study to evaluate the correlation between the nutritional status and systemic inflammation in patients with CRE. Although some inspiring results were obtained, there were some notified limitations. Firstly, BIA was only implemented at one time point before nutritional intervention was initiated and should be implemented at multiple time points to provide thorough tutorial suggestions for clinicians. Secondly, possible selection bias of the convenience subjects was introduced by excluding patients those who couldn’t complete these methods or questionnaire.

It is recommended that patients with CRE should undergo different nutritional assessments, including BIS combined with nutritional assessments and biochemical markers. Accordingly, appropriate strategies for nutritional support could be designed before implementing any interventions. Afterwards, re-evaluation of the nutritional and inflammatory status after a period of nutritional intervention and adjustments of further improved nutrition managements are necessary.

## Conclusion

The present study showed that most patients with CRE suffered from malnutrition. It was intuitive to notice that these patients were thinner and had significantly lower body composition parameters compared with controls. It is concluded that nutritional assessments combined with BIS and biochemical markers are appropriate methods to assess the nutritional and inflammatory status in patients with CRE. Moreover, this study revealed that the nutritional status was significantly associated with systemic inflammation. Further researches regarding correlations of the nutritional status with complications and mortality need to be investigated in a prospective study.

## Abbreviations

BCM, Body cell mass; BIA, Bioelectrical impedance analysis; BIS, Bioelectrical impedance spectroscopy; CONUT, Controlling Nutritional Status; CRE, Chronic radiation enteritis; CRP, C-reactive protein; ECW, Extracellular water; FFM, Fat-free mass; FM, Fat mass; ICW, Intracellular water; NLR, Neutrophil/lymphocyte ratio; NRS-2002, Nutritional Risk Screening-2002; PG-SGA, Patient-Generated Subjective Global Assessment; TBW, Total body water; TC, Total cholesterol
